# Supplementary material for: Rodents as Hosts of Pathogens and Related Zoonotic Disease Risk
Source: Pathogens. 2020 Mar 10;9(3):202. doi: 10.3390/pathogens9030202 (PMC7157691; doi:10.3390/pathogens9030202)
Supplement: Supplementary file 1 [file pathogens-09-00202-s001.pdf]

Supplementary documents:

Table S1. The list of negative and positive DNA controls, used to confirm the sensitivity and specificity of the PCR systems designed for this study.

**Microorganism**	**Name**	**Origin**
Bacteria	*Staphylococcus haemolyticus*	Laboratory colony
Bacteria	*Staphylococcus aureus*	Laboratory colony
Bacteria	*Rickettsia felis*	Laboratory colony
Bacteria	*Wolbachia* PL13	Laboratory colony
Bacteria	*Stenotrophomonas maltophilia*	Laboratory colony
Bacteria	*Acinetobacter* sp.	Laboratory colony
Bacteria	*Enterobacter aerogenes*	Laboratory colony
Bacteria	*Yersinia pestis*	Laboratory colony
Bacteria	*Rickettsia montanensis*	Laboratory colony
Lice	Head lice *(Pediculus humanus capitis)*	Homo sapiens (Amazonia)
Lice	*Pediculus humanus*	Laboratory breeding
Bacteria	*Ehrlichia*	Laboratory colony
Bacteria	*Coxiella burnetii*	Laboratory colony
Bacteria	*Borrelia recurrentis*	Laboratory colony
Bacteria	*Staphylococcus hominis*	Laboratory colony
Bacteria	*Asaia bogorensis*	Laboratory colony
Bacteria	*Haemophilus influenzae*	Laboratory colony
Bacteria	*Wolbachia*	Laboratory colony
Bacteria	*Anaplasma phagocytophilum*	Laboratory colony
Bacteria	*Enterobacter aerogenes*	Laboratory colony
Bacteria	*Acinetobacter baumannii*	Laboratory colony

Bacteria	*Streptococcus pneumoniae*	Laboratory colony
Bacteria	*Salmonella enterica*	Laboratory colony
Bacteria	*Citrobacter koseri*	Laboratory colony
Bacteria	*Gardnerella vaginalis*	Laboratory colony
Bacteria	*Streptococcus pyogenes*	Laboratory colony
Parasite	*Plasmodium falciparum*	Laboratory colony
Bacteria	*Rickettsia typhi*	Laboratory colony
Bacteria	*Enterococcus faecium*	Laboratory colony
Bacteria	*Streptococcus agalactiae*	Laboratory colony
Bacteria	*Rickettsia conorii*	Laboratory colony
Bacteria	*Asaia bogorensis*	Laboratory colony
Bacteria	*Bacillus thuringiensis*	Laboratory colony
Tick	*Amblyomma variegatum*	Senegal
Dog	DH62 cell line	Cell line
Tick	BME (*Rhipicephalus microplus*)	Cell line
Horse	CV.G 22 (*Equus caballus*)	French Guiana
Donkey	ANE 4 (*Equus asinus*)	Egypt
Human	HL60 cell line (*Homo sapiens*)	Cell line
Mouse	L929 cell line	Cell line
Flea	*Ctenocephalides felis*	Laboratory colony
Bedbugs	*Cimex lectularius*	Laboratory colony
Tick	*Hyalomma marginatum*	Senegal
Bacteria	*Bartonella henselae*	Laboratory colony
Bacteria	*Bartonella* sp.	Laboratory colony
Bacteria	*Rickettsia canadensis*	Laboratory colony
Bacteria	*Bartonella quintana*	Laboratory colony

Bacteria	*Rickettsia australis*	Laboratory colony
Bacteria	*Borrelia crocedurae*	Laboratory colony
Parasite	*Hepatozoon canis*	Laboratory colony
Parasite	*Dirofilaria immitis*	Laboratory colony
Parasite	*Trypanosoma evansi*	Laboratory colony
Parasite	*Leishmania*	Laboratory colony
Parasite	*Leishmania major*	Laboratory colony
Parasite	*Trypanosoma congolense*	Laboratory colony
Parasite	*Trypanosoma gambiense*	Laboratory colony
Bacteria	*Streptobacillus moniliformis*	Laboratory colony
Bacteria	*Borrelia theileri*	Laboratory colony
Bacteria	*Borrrelia* sp.	Laboratory colony
Bacteria	*Vibrio cholerae*	Laboratory colony
Bacteria	*Treponema pallidum*	Laboratory colony
